# Clinical and Mortality Risk Factors in Bloodstream Infections with Carbapenem-Resistant Enterobacteriaceae

**DOI:** 10.1155/2017/6212910

**Published:** 2017-12-12

**Authors:** Xiaopeng Li, Huan Ye

**Affiliations:** Department of Infectious Diseases, Fuxing Hospital, Capital Medical University, Beijing, China

## Abstract

**Objective:**

To investigate the risk factors underlying the occurrence and mortality of bloodstream infections (BSIs) with carbapenem-resistant Enterobacteriaceae (CRE).

**Methods:**

Medical information was retrospectively analyzed from 148 cases of patients with Enterobacteriaceae BSIs at a medical center in China, between 2013 and 2015.

**Results:**

The 30-day mortality rate in the CRE group was 65.4%. Indwelling urethral catheterization, admission to the ICU, use of antibiotics within 30 days, and BSIs from the respiratory system were associated with CRE BSIs. Lung infection, abdominal infection, central venous catheterization, and use of hormones within 30 days were associated with mortality.

**Conclusion:**

The 30-day mortality rate of CRE BSIs was high. Lung infections, abdominal infections, central venous catheterization, and use of hormones within 30 days increased the mortality rate of Enterobacteriaceae BSIs.

## 1. Introduction

Recently, infections with drug-resistant bacterial strains have increased which poses a challenge for anti-infection treatments in the clinical context and has become a serious problem in the field of public health. The incidence of bloodstream infections is also increasing and has become a major cause of the occurrence of infectious diseases and deaths worldwide [1]. Bloodstream infections with multidrug-resistant Enterobacteriaceae account for 71.5% of all bloodstream infections with multidrug-resistant bacterial strains [2]. Carbapenem antibiotics are typically one of the most effective drug classes for the treatment of Enterobacteriaceae infections. However, carbapenem-resistant Enterobacteriaceae (CRE) has been identified recently within all Enterobacteriaceae spp. The CHINET monitoring results show that *Klebsiella pneumoniae* accounts for the majority of cases of CRE infections among the Enterobacteriaceae spp., with drug resistance rates to imipenem and meropenem > 10% [3]. Studies have shown that the mortality rate is significantly higher for CRE infections than for carbapenem-sensitive Enterobacteriaceae infections [4].

This study was a retrospective analysis of medical information on bloodstream infections with Enterobacteriaceae (mainly *Klebsiella pneumoniae*, *Escherichia coli*, *Enterobacter cloacae*, and *Enterobacter aerogenes*) with intent to identify the risk factors and prognosis for CRE bloodstream infections.

## 2. Materials and Methods

### 2.1. Study Subjects

A total of 148 cases of bloodstream infections with Enterobacteriaceae that occurred during the period between January 2013 and October 2015 in Fuxing Hospital of Capital Medical University, China, were selected. Clinical information from the patients, including demographic data, underlying diseases, pathogen drug resistance and drug sensitivity analyses, history of medical institution admission, level of care (ward versus ICU), history of invasive surgery, and prognosis, was recorded and analyzed. The characteristics of each case were collected from the electronic medical record database, and the files of all patients were complete.

Inclusion criteria: On the day of collection of a positive blood culture specimen, the patient had a temperature ≥ 38°C or < 36°C in combination with chills, and the multiple blood culture results showed the same type of bacteria. Exclusion criteria: (1) blood culture test results showing two or more types of bacteria and (2) only one blood culture specimen was positive, whereas multiple subsequent blood culture results were negative or identified other types of pathogens.

This study conformed to the standards of medical ethics and was approved by the Ethics Committee of Fuxing Hospital of Capital Medical University.

### 2.2. Relevant Definitions

The microbiology laboratory in our hospital currently uses an FX200 automatic blood culture instrument (BACTE) and a WalkAway 40 automatic microbiological drug sensitivity analysis instrument (USA). The drug sensitivity results were determined according to the Clinical and Laboratory Standards Institute (CLSI, USA) standards [5]. The definition of CRE was resistance to imipenem, meropenem, or ertapenem [3]. Extensively drug-resistant (XDR) bacteria were defined based on nonsusceptibility to all antimicrobial agents except colistin and tigecycline [6, 7]. Long-term bedbound patients were defined as patients who were bedbound for more than 14 days and could not recover [7].

### 2.3. Statistical Methods

The data processing and analysis were performed using SPSS 21.0 statistical software. *P* < 0.05 indicated that the difference was significant. Measurement data with a normal distribution are expressed using the mean ± standard deviation. Measurement data with a nonnormal distribution are expressed using the median. Significance testing for differences between groups was performed using the *t*-test (normal distribution) or rank sum test (nonnormal distribution). Significance testing for differences in count data between groups was performed using the *χ*^2^ test. The multivariate analysis was performed using logistic regression.

## 3. Results

### 3.1. Demographic Data and Clinical Characteristics

This study involved a total of 148 cases of bloodstream infections with Enterobacteriaceae. The mean age of the patients was 69.1 (±19.2) years. Male patients accounted for 50.7% of the cases, and 20.3% of the patients were admitted to the ICU. Long-term bedbound patients accounted for 54.1% of the cases, and 62.2% of the patients had a medical institution admission history within 3 months. The bloodstream infections mainly originated from the urinary tract (28.4%) or respiratory system (25.0%) or were catheter related (22.3%). Patients who had prior use of broad-spectrum antibiotics within 30 days accounted for 43.2% of the cases. The total average length of hospital stay was 21.7 (±12.5) days. Patients with CRE infections accounted for 17.6% of the cases, and patients with XDR Enterobacteriaceae infections accounted for 4.0% of the cases.

The results of the univariate and multivariate analyses of risk factors related to CRE bloodstream infections are shown in Tables 1 and 2, respectively (the relevant factors listed in the tables were all conditions before the presence of the bloodstream infection). The logistic regression analysis revealed that indwelling urethral catheterization, admission to the ICU, CRE infection of the respiratory system, and prior use of broad-spectrum antibiotics within 30 days were independent risk factors for CRE bloodstream infections.

### 3.2. Analysis of Risk Factors for Mortality in Bloodstream Infections with Enterobacteriaceae

According to the comparison of the 30-day prognosis between groups, the 30-day mortality rate for bloodstream infections with Enterobacteriaceae was 25.7%. Univariate analysis indicated that community acquired, admission to the ICU, long-term bedbound, anemia, hypoalbuminemia, lung infection, abdominal infection, liver dysfunction, mechanic ventilation, central venous catheterization, indwelling urethral catheterization, sources of bloodstream infection from catheter related and urinary tract, antibiotics within 30 days, combined fungal infection, prior use of glucocorticoids within 30 days, and CRE infection were significantly associated with 30-day risk of mortality (Tables 3). A logistic regression analysis revealed that lung infection, abdominal infection, central venous catheterization, and prior use of glucocorticoids within 30 days were independently associated with mortality (Tables 4).

## 4. Discussion

Currently, the incidence of carbapenemase-producing Enterobacteriaceae is increasing. Data have shown that many countries have interregional spread or an endemic situation [8]. The results of this study showed that the 30-day mortality rate for CRE bloodstream infections reached 65.4%, which was significantly higher than the rate for non-CRE bloodstream infections (17.2%) or for CRE-infected patients in literature reports (26%–44%) [4]. To date, many studies have evaluated risk factors related to bloodstream infections with multidrug-resistant Enterobacteriaceae. For example, factors including admission to the ICU, length of hospital stay, history of use of broad-spectrum antibiotics, such as quinolones and cephalosporins, history of resistant strain colonization, indwelling urethral catheterization, and central venous catheterization have been considered independent risk factors for bloodstream infections with multidrug-resistant Enterobacteriaceae [4, 9]. The analytical results in this study showed that indwelling urethral catheterization, admission to the ICU, prior use of broad-spectrum antibiotics within 30 days, and CRE infection of the respiratory system were independent risk factors for CRE bloodstream infections. The first three factors are similar to previous reports. However, what is different from previous reports is that we identified CRE infection of the respiratory system as an independent risk factor for CRE bloodstream infections. Respiratory tract infection was the most common source of bloodstream infection in our study, likely because of a decreased capacity for bacterial clearance resulting from the altered lung tissue in severe chronic obstructive lung disease.

The results of this study on risk factors for death due to bloodstream infections with Enterobacteriaceae showed that lung infections, abdominal infections, central venous catheterization, and hormone use within 30 days were all independent risk factors for death due to bloodstream infections with Enterobacteriaceae. The study results showed that the mortality rate was significantly higher in the CRE group than in the non-CRE group. The high mortality associated with CRE bloodstream infections was partially attributed to host conditions, such as endogenous infections and invasive surgery. Additionally, the mortality rate was associated with a lack of timely and accurate antibiotic treatment for infection with drug-resistant bacterial strains or even no antibiotic treatment [10]. XDR strains accounted for 23% of the 26 cases of CRE infections in this study, which was similar to the 28% rate of XDR bacterial strains isolated from CRE infections reported in the literature [8]. This result indicated that approximately one-fourth of CRE infections might be XDR, which is associated with a high mortality rate.

During the analysis of mortality-related risk factors in patients with bloodstream Enterobacteriaceae infections in this study, CRE infection was a relevant but not an independent risk factor, which might be associated with the low overall number of cases.

In summary, CRE bloodstream infections have a high mortality rate, and treatment has poor clinical efficacy. The use of broad-spectrum antibiotics, indwelling urethral catheterization, admission to the ICU, and infection from the respiratory system were independent risk factors for CRE bloodstream infections. Lung infections, abdominal infections, hormone use, and central venous catheterization were independent risk factors for death in patients with Enterobacteriaceae bloodstream infections. Therefore, the standard use of antibiotics, minimization of invasive operations, use of strict aseptic manipulation, and strengthening of hand-hygiene awareness of healthcare workers may be effective measures for the prevention of CRE bloodstream infections.

## Figures and Tables

**Table 1:** Univariate analysis of the clinical characteristics of bloodstream infections caused by CRE and non-CRE.

Clinical characteristics	Bloodstream infections with Enterobacteriaceae, *n* (%)		*P* value
	CRE group (*n* = 26)	Non-CRE group (*n* = 122)	
Gender (male)	16 (61.5)	59 (48.4)	0.222
Age > 60 years	22 (84.6)	83 (68.0)	0.091
Community acquired	0	30 (24.6)	0.005^∗^
Admission to the ICU	20 (76.9)	10 (8.2)	<0.001^∗^
Long-term bedbound	19 (73.1)	61 (50)	0.032^∗^
Medical institution admission history within 3 months	17 (65.4)	75 (61.5)	0.709
Underlying diseases			
Cardiovascular disease	19 (73.1)	54 (44.3)	0.008^∗^
Cerebrovascular disease	21 (80.8)	57 (46.7)	0.002^∗^
Chronic respiratory disease	6 (23.1)	9 (7.4)	0.040^∗^
Chronic kidney disease	13 (50.0)	33 (27.0)	0.022^∗^
Diabetes mellitus	9 (34.6)	44 (36.1)	0.889
Malignant tumor	4 (15.4)	44 (36.1)	0.041^∗^
Anemia	26 (100.0)	87 (71.3)	0.002^∗^
Hypoalbuminemia	26 (100.0)	98 (80.3)	0.029^∗^
Lung infection	22 (84.6)	61 (50.0)	0.001^∗^
Urinary tract infection	12 (46.2)	62 (50.8)	0.666
Abdominal infection	5 (19.2)	14 (11.5)	0.453
Tracheotomy	12 (46.2)	21 (17.2)	0.001^∗^
Liver dysfunction	13 (50.0)	24 (19.7)	0.001
Invasive surgery before infection			
Mechanical ventilation	18 (69.2)	12 (9.8)	<0.001^∗^
Central venous catheterization	19 (73.1)	28 (23.0)	<0.001^∗^
Indwelling urethral catheterization	25 (96.2)	45 (36.9)	<0.001^∗^
Sources of bloodstream infection			
Catheter related	10 (38.5)	23 (18.9)	0.029^∗^
Respiratory system	11 (42.3)	26 (21.3)	0.025^∗^
Urinary tract infection	0 (0)	42 (34.4)	<0.001^∗^
Others	5 (19.2)	31 (25.4)	0.505
Prior use of broad-spectrum antibiotics within 30 days	25 (96.2)	39 (32.0)	<0.001^∗^
Combined fungal infection	10 (38.5)	10 (8.2)	<0.001^∗^
Prior use of glucocorticoids within 30 days	8 (30.8)	11 (9.0)	0.007^∗^
Total length of hospital stay	25.4 ± 11.1	21.0 ± 12.7	0.102
30-Day mortality rate	17 (65.4)	21 (17.2)	<0.001^∗^
48-Hour mortality rate	6 (23.1)	3 (2.5)	<0.001^∗^

ICU: intensive care unit; ^∗^*P* < 0.05.

**Table 2:** Analysis of risk factors for CRE bloodstream infections (logistic regression analysis).

Risk factor	OR value	95% CI	*P* value
Indwelling urethral catheterization	11.40	1.05 to 124.18	0.046
Admission to the ICU	9.42	2.14 to 41.50	0.003
Respiratory system source	8.95	1.73 to 46.38	0.009
Prior use of broad-spectrum antibiotics within 30 days	11.25	1.19 to 106.79	0.035

OR: odds ratio; 95% CI: 95% confidence interval; respiratory system source: bloodstream infection with Enterobacteriaceae from the respiratory system.

**Table 3:** Univariate analysis for predictors of mortality caused by bloodstream infections with Enterobacteriaceae.

Clinical characteristics	Bloodstream infections with Enterobacteriaceae, *n* (%)		*P* value
	Survival group (*n* = 110)	Mortality group (*n* = 38)	
Gender (male)	55 (50.0)	20 (52.6)	0.780
Age	67.5 ± 20.0	73.9 ± 15.9	0.075
Community acquired	28 (25.5)	2 (5.3)	0.008^∗^
Admission to the ICU	10 (9.1)	20 (52.6)	<0.001^∗^
Long-term bedbound	50 (45.5)	30 (78.9)	<0.001^∗^
Medical institution admission history within 3 months	69 (62.7)	23 (60.5)	0.809
Underlying diseases			
Cardiovascular disease	51 (46.4)	22 (57.9)	0.220
Cerebrovascular disease	54 (49.1)	24 (63.2)	0.134
Chronic respiratory disease	12 (10.9)	3 (7.9)	0.827
Chronic kidney disease	30 (27.3)	16 (42.1)	0.089
Diabetes mellitus	38 (34.5)	15 (39.5)	0.585
Malignant tumor	37 (33.6)	11 (28.9)	0.594
Anemia	77 (70.0)	36 (94.7)	0.002^∗^
Hypoalbuminemia	87 (79.1)	37 (97.4)	0.008^∗^
Lung infection	53 (48.2)	30 (78.9)	0.001^∗^
Urinary tract infection	60 (54.5)	14 (36.8)	0.060
Abdominal infection	9 (8.2)	10 (26.3)	0.009^∗^
Tracheotomy	22 (20.0)	11 (28.9)	0.253
Liver dysfunction	20 (18.2)	17 (44.7)	0.001^∗^
Invasive surgery before infection			
Mechanic ventilation	10 (9.1)	20 (52.6)	<0.001^∗^
Central venous catheterization	20 (18.2)	27 (71.1)	<0.001^∗^
Indwelling urethral catheterization	41 (37.3)	29 (76.3)	<0.001^∗^
Sources of bloodstream infection			
Catheter related	19 (17.3)	14 (36.8)	0.012^∗^
Respiratory system	25 (22.7)	12 (31.6)	0.277
Urinary tract	39 (35.5)	3 (7.9)	0.001^∗^
Others	27 (24.5)	9 (23.7)	0.915
Prior use of broad-spectrum antibiotics within 30 days	36 (32.7)	28 (73.7)	<0.001^∗^
Combined fungal infection	9 (8.2)	11 (28.9)	<0.001^∗^
Prior use of glucocorticoids within 30 days	4 (3.6)	15 (39.5)	0.007^∗^
CRE infection	9 (8.2)	17 (44.7)	<0.001^∗^
Total length of hospital stay	21.4 ± 13.2	22.7 ± 10.4	0.528

CRE: carbapenem-resistant Enterobacteriaceae; ^∗^*P* < 0.05.

**Table 4:** Logistic regression analysis of risk factors for mortality in patients with Enterobacteriaceae bloodstream infections.

Risk factor	OR value	95% CI	*P* value
Lung infection	6.62	1.86 to 23.61	0.004
Abdominal infection	11.15	2.54 to 48.92	0.001
Central venous catheterization	5.25	1.84 to 14.94	0.002
Prior use of glucocorticoids within 30 days	22.40	4.07 to 123.38	<0.001

OR: odds ratio; 95% CI: 95% confidence interval.
